# Deploying mutation impact text-mining software with the SADI Semantic Web Services framework

**DOI:** 10.1186/1471-2105-12-S4-S6

**Published:** 2011-07-05

**Authors:** Alexandre Riazanov, Jonas Bergman Laurila, Christopher JO Baker

**Affiliations:** 1 Department of Computer Science & Applied Statistics, University of New Brunswick, Saint John, New Brunswick, E2L 4L5, Canada

## Abstract

**Background:**

Mutation impact extraction is an important task designed to harvest relevant annotations from scientific documents for reuse in multiple contexts. Our previous work on text mining for mutation impacts resulted in (i) the development of a GATE-based pipeline that mines texts for information about impacts of mutations on proteins, (ii) the population of this information into our OWL DL mutation impact ontology, and (iii) establishing an experimental semantic database for storing the results of text mining.

**Results:**

This article explores the possibility of using the SADI framework as a medium for publishing our mutation impact software and data. SADI is a set of conventions for creating web services with semantic descriptions that facilitate automatic discovery and orchestration. We describe a case study exploring and demonstrating the utility of the SADI approach in our context. We describe several SADI services we created based on our text mining API and data, and demonstrate how they can be used in a number of biologically meaningful scenarios through a SPARQL interface (SHARE) to SADI services. In all cases we pay special attention to the integration of mutation impact services with external SADI services providing information about related biological entities, such as proteins, pathways, and drugs.

**Conclusion:**

We have identified that SADI provides an effective way of exposing our mutation impact data such that it can be leveraged by a variety of stakeholders in multiple use cases. The solutions we provide for our use cases can serve as examples to potential SADI adopters trying to solve similar integration problems.

## Background

The annotation of mutants with their consequences is central task for researchers investigating the role of genetic changes on biological systems and organisms. These annotations facilitate the reuse and reinterpretation of mutations and are necessary for the establishment of a comprehensive understanding of genetic mechanisms, biological processes and the resulting mutant phenotypes. As a result, there are numerous mutation databases, albeit perpetually out of date and often with a latency of many years, which is an instance of the general latency problem with genomic and proteomic databases [1]. Automated mutation extraction systems based on text mining techniques can identify and deliver mutation annotations for database curators to review, or directly to end users. In this article we outline the publication of a mutation impact extraction system in the form of semantic web services, and their integration with other semantically described bioinformatics services, based on the SADI framework.

In our previous work we developed the Mutation Impact pipeline [2] - a program, based on a GATE [3] pipeline, that makes it possible to extract mutation impacts on protein properties from texts, categorising the directionality of impacts as positive, negative or neutral. Moreover, the system maps mentions of proteins and mutations to their respective UniProt identifiers and protein properties described in the Gene Ontology.

For example, consider these two excerpts from [4]: “The *haloalkane dehalogenase* from the nitrogen-fixing hydrogen bacterium *Xanthobacter autotrophics* GJ10 (Dh1A) prefers 1,2-dichloroethane (DCE) as substrate and converts it to 2-chloroethanol and chloride” and “Dh1A shows only a small *decrease in activity* when *Trp-125 is replaced with phenylalanine*”*.* Our pipeline (i) identified “*haloalkane dehalogenase*” as a protein, (ii) mapped it to the UniProt ID P22643 by grounding it to the identified organism “*Xanthobacter autotrophicus*”, (iii) identified “*Trp-125 is replaced with phenylalanine*” as the point mutation W125F, (iv) identified “*activity*” as a protein property (GO_00188786 in the Gene Ontology, and (v) identified “*decrease*” as the direction of the impact of the mutation on the protein property.

Initially, the Mutation Impact pipeline was deployed as a simple Java API and could only be used programmatically. When the pipeline is executed on a document, it computes a sequence of Java objects representing mutation specifications. Every such object contains information about a series of elementary mutations that are studied together, the corresponding wildtype and mutant proteins, and the discovered impacts of the mutations. The Java object representing an impact contains the direction of the impact, e.g., positive, negative or neutral, and the type of the protein property being affected as a Gene Ontology term ID, e.g., “GO_00188786”.

Although the practical use of the system and its results in this form is maximally flexible and may be preferred by many programmers, having some programming-free modes of use, e.g., based on Semantic Web standards, could extend the usability. So in [2] we explored the possibility of using semantic technologies for exporting the text mining pipeline outputs according to a domain specific knowledge representation. Currently, our system, like mSTRAP [5], delivers its results in the form of an OWL ABox, i.e., as a collection of logical statements characterising the extracted mutations, proteins and impacts. The classes and property predicates in these statements are defined in our Mutation Impact ontology [6] in OWL, based on the earlier mutation ontology from [7]. The ontology is briefly described in the Methods section.

Representing text mining results as class and property assertions with respect to the Mutation Impact ontology already adds a great deal of flexibility – the results can be used with any toolsets that work with OWL. The most straightforward way of using semantically described data is by querying it directly, so we established a semantic database, in the form of a Sesame [8] RDF triplestore, that stores the results of mining different documents. For our experiments, the database is populated with mutation information extracted from 756 journal articles, with 2993 extracted mentions of point mutations and 519 extracted mentions of mutation impacts on protein properties of 116 distinct types. Our users can query the populated database via a SPARQL [9] end-point [10]. Since we keep the links from the extracted entities and associations to the corresponding publications, the database can also be considered a form of semantic index for texts.

As we would like to facilitate a multitude of data reuse cases, the provision of a SPARQL endpoint as the sole data access form is not sufficient. Consequently, we are looking for additional ways to provide access to the data. Our primary requirement is that the framework should support integration with other software and data for proteins, mutations, impacts and related biological entities, such as pathways, and drugs. This criterion is important because isolated mutation impact mining results have limited reusability outside the domain of protein engineering.

In this article we review the SADI framework [11,12] as a candidate platform for providing access to our semantically exposed mutation impact data. The choice is based on the powerful integrative features displayed by SADI services and client software, discussed in the next section. This article describes an *exploratory case study* using five biologically meaningful queries that require (i) some data from our text mining pipeline and the Mutation Impact DB, as well as (ii) some biological knowledge from external sources. Furthermore, we test the queries using the SHARE client [13] which is designed to automatically discover and combine the required SADI services.

The work presented here is a part of a bigger effort: by doing extensive coherent case studies with SADI in several biomedical domains we are (i) developing a transferable methodology in the form of best practices and recipes covering typical problems, so that future SADI adopters can copy existing solutions and adapt them to their needs, and (ii) trying to learn the extent of the capabilities and the soft spots of the SADI framework in the hope that this will help the future development of SADI and related Semantic Web Services techniques. As a valuable byproduct of the case study presented here, we created a prototype semantic infrastructure that provides the flexibility required by multiple uses of our mutation mining software and the Mutation Impact DB.

## Methods

### What is SADI?

The SADI framework [11,12] is a set of conventions for creating Semantic Web Services that can be *automatically discovered and orchestrated*. A SADI-compliant service consumes a whole RDF document as input and produces an RDF document as output. This convention alone eliminates the problem of syntactic interoperability because all SADI services “speak” the same language. This is also convenient for client programs that can leverage existing APIs for RDF to represent the data on which SADI services operate.

An input RDF document has some URI node designated as the *central input node*, and the whole input graph is considered a description of the central node. Exactly the same URI is always present in the output graph as the central output node. The sole function of a SADI service is to *annotate this node with new properties* and assert these properties in the output RDF document, in contrast with more conventional Web services that usually compute output without an explicit connection to the input.

The most important feature of SADI is that the predicates for these property assertions are fixed for each service. A declaration of these predicates, available online, constitutes a *semantic description* of the service. For example, if a service is declared with the predicate *myontology*:*isTargetOfDrug* described in an ontology as a predicate linking proteins to drugs, the user knows that he can use the service to search for drugs targeting a given protein.

The declaration of the service predicates is done by specifying an OWL class for the output nodes. If this *output class* entails an *existential restriction* for some predicate *R*, i.e., it is postulated that every instance of the output class is linked with *R* to some entity, it means that the predicate is declared to be produced by the service and the corresponding output data may be available from the service. Registries of SADI services can use such predicates to index the services providing them, thus enabling *service discovery based on required functionality.*

Another part of a service declaration is the input (OWL) class that imposes restrictions on the kind of input URIs the service can process. In particular, if this class subsumes an intersection of property restrictions, a well-behaved service will look for the corresponding properties attached to an input node, and use the values as parts of the input.

As an example, consider the SADI service [14] computing the Body Mass Index of a person, which is defined as the person’s weight divided by the square of the persons height. Its *InputClass* is defined as the intersection of *mged*:*has_height ***some*** mged*:*Measurement* and *mged*:*has_mass ***some*** mged*:*Measurement*, in Manchester Syntax [15] (for the meaning of frequently used URI prefix abbreviations like *mged* the reader is referred to Table 1), so the service expects the property predicates *mged*:*hasheight* and *mged*:*hasjmass* attached to an input node. The service’s *OutputClass* is a subclass of *bmi*:*BMI*** some ***xs*:*int*, so the service provides the predicate *bmi*:*BMI* (*bmi* corresponds to the service’s own ontology that describes the input and output classes). Given the following RDF (presented here in the Notation 3 syntax [16] for readability) as input

**Table 1:** URI prefixes used in the paper

abbreviation	URI prefix
bibo	http://purl.org/ontology/bibo/
dbsnp	http://lsrn.org/dbSNP:
dc	http://purl.org/dc/elements/1.1/
foaf	http://xmlns.com/foaf/0.1/
go	http://purl.org/obo/owl/GO#
lsrn	http://purl.oclc.org/SADI/LSRN/
mged	http://mged.sourceforge.net/ontologies/MGEDOntology.owl#
mio	http://unbsj.biordf.net/ontologies/mutation-impact-ontology.owl#
mioe	http://unbsj.biordf.net/ontologies/mutation-impact-ontology-extras.owl#
mis	http://unbsj.biordf.net/mutation-impact/mi-sadi-service-ontology.owl#
mms	http://www.mygrid.org.uk/mygrid-moby-service#
obj	http://sadiframework.org/ontologies/service_objects.owl#
owl	http://www.w3.org/2002/07/owl#
pmc	http://www.ncbi.nlm.nih.gov/pmc/articles/PMC/
pred	http://sadiframework.org/ontologies/predicates.owl#
props	http://sadiframework.org/ontologies/properties.owl#
rss	http://purl.org/rss/1.0/
sadiont	http://sadiframework.org/ontologies/sadi.owl#
sio	http://semanticscience.org/resource/
uniprot	http://biordf.net/moby/UniProt/

the service generates this RDF as output:

The declaration of the input and output classes of a SADI service constitutes a *semantic description* of the service. Importantly, such semantic descriptions allow completely automatic discovery and composition of SADI services (see, e.g., [11,13]). In our settings, using SADI services to provide access to the Mutation Pipeline and DB will allow automatic integration with hundreds of external databases and programs dealing with mutations, proteins and related biomedical entities, e.g., pathways and drugs, so long as there are SADI services for these resourses. These are desirable features of SADI motivating us to deploy our mutation impact software with this framework.

Finally, let us mention some important technicalities. SADI services are defined on top of the HTTP protocol. A SADI service is requiredto implement HTTP GET and POST. A valid response to an HTTP GET is a description of the service in RDF. It specifies the input and output classes and provides some additional information about the service, such as a brief textual description. The class URIs must resolve to the corresponding OWL ontology files. Service invocation is done with POST: the client sends the input RDF document as the content of a POST message, and the service returns the output RDF graph in the response. It is convenient to implement such services using standard Java servlets which are supported by a number of robust server implementations, e.g., Apache Tomcat. For greater convenience, the SADI framework provides a Java API that specialises javax.servlet.Servlet so that the SADI service programmer only needs to deal with RDF in the input and the output. A similar Perl library also exists.

### SHARE: a SPARQL engine for SADI services

SHARE [13] is an experimental client featuring automatic discovery and orchestration of SADI services. From the user point of view, SHARE is a SPARQL engine that computes queries by picking and calling suitable SADI services from some registry. In a typical scenario, the user first looks up predicates he needs for his query, in the list of predicates declared as provided by SADI services in a registry, and also related classes and property predicates in the referenced ontologies. Then he uses the available concepts to form a regular SPARQL query, and sends it to a SHARE endpoint. Importantly, the SHARE engine decides itself which services have to be invoked and in what order, to execute the query. Note that this qualifies for *automatic discovery*, *composition and invocation*. The user deals only with an almost declarative query, i.e., he only needs to understand the semantics of the URIs being used in the query, although knowing the services providing the predicates can be beneficial. This situation suits our purposes well, so, for our experiments with SADI services for Mutation Impact data we are using the Web interface for SHARE [17].

To have a controlled environment for our experiments, we installed SHARE (see [18]) on our own server – a QuadCore 1.8 MHz PC with large cache and RAM, running Ubuntu Linux, together with a local installation of a SADI registry that only contains services relevant to this case study. Relying on our experience, we recommend this way of doing large case studies because having a local SHARE installation allows to debug queries by analysing SHARE logs, and also makes the experiments reproducible regardless of the changes in the public registry or the SHARE code. Note that although our services are accessible from both the central SHARE installation and our local one, the results and performance of queries on the two installations may differ significantly because the registry used by the central SHARE installation contains a much bigger number of different services. The SHARE client is still in its infancy and makes some redundant service calls in presence of many registered services. Although we provide some performance figures, such as the numbers of found answers and execution times for some of our queries, at this stage the query performance is not a concern for us since we are only investigating the general applicability of the SADI framework to our use cases.

Since SHARE is just a SPARQL engine, its effective use is highly dependent on the ability of users to write meaningful queries. To write queries that can be executed, users need to know what classes and property predicates are available, i. e., what predicates are provided by the registered services and what classes and predicates are axiomatically related to them in the corresponding ontologies. Currently, the main way of listing predicates provided by the services in a registry is to query the SPARQL endpoint associated with the registry. For example, the central public SADI registry [19] has a SPARQL endpoint http://sadiframework.org/registry/sparql/, and querying this endpoint with

will produce a list of services with the predicates they provide (as well as the services’ textual descriptions). Note that the query uses some prefixes defined in Table 1. Currently, there is no support for retrieving entities related to these predicates via the corresponding ontologies, e. g., inverse predicates, so this kind of search has to be done manually. In many cases, although not always, the predicate URIs are resolved to files with the ontologies defining them, and related entities can be found by examining these ontologies.

Another SHARE-related limitation stems from the fact that the current implementation does not guarantee completeness – some answers that can be computed in principle, won’t be found by the system. This is, however, *not an inherent problem* for SADI as there likely to exist query client architectures with completeness guarantee, although without a termination guarantee, since complete sets of answers may be infinite.

### Mutation Impact Ontology

**Figure 1:**
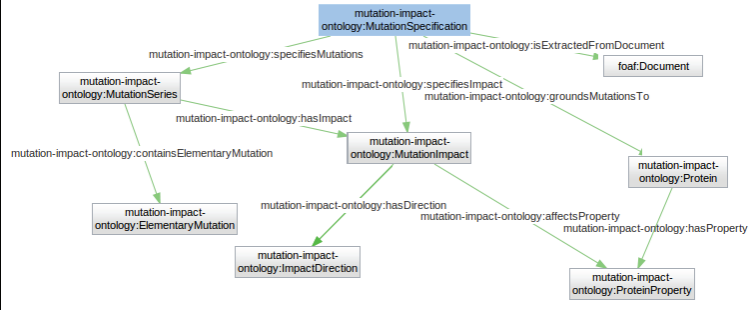
**Mutation impact ontology structure**. Visualization of top level concepts as *Mutation Specification*, *Protein*, *Mutation Impact* and *Protein Property* being connected through object property predicates.

Since the SADI services based on our text mining software are defined in terms of our Mutation Impact ontology, we would like to give a brief overview of the ontology here. Figure 1 shows the top level concepts of the ontology with some relations between them.

The central concept in our ontology is mutation specification. Intuitively, an instance of this class is a piece of information or a statement saying that some mutation applied to a specified protein has a specified impact on a specified protein property. There are, correspondingly, classes representing mutations (more specifically, series of elementary mutations), proteins, protein properties and impacts.

The main predicates relating these classes are as follows. The predicate *specifiesMutations* links to the mutation series that a mutation specification describes. The membership of elementary mutations in mutation series is expressed with the predicate *containsElementaryMutation*. The wildtype protein is specified with *groundMutationsTo* and the impact is specified with *specifiesImpact*. An instance of impact is characterised with its direction, e.g., positive, negative or neutral, via *hasDirection*, and with an instance of the affected protein property. Note that protein properties are also modelled as individuals. They can be instances of different subclasses of *ProteinProperty* – currently we use the Gene Ontology classes for molecular functions. Protein properties are grounded to proteins: apart from the protein property class, a specific protein is assigned to a property instance with the predicate *hasProperty*. Since our ontology is mainly aimed at representing text mining results, mutation specification instances are linked to the documents they are extracted from with the predicate *isExtractedFromDocument*, which is a subproperty of the inverse of *foaf:topic*. This FOAF [20] predicate can be interpreted as having a slightly stronger semantics than necessary for our purposes because its description “a topic of some page or document” can be interpreted as “the main topic of some page or document” by some users. However, we failed to find a better predicate in a sufficiently standard vocabulary. Currently, we are using *foaf:topic* in parallel with the SIO [21] predicate *’refers to’* (*SIO_*000628) with a more precise semantics, and in the future it may completely replace *foaf:topic.*

In addition to the object property predicates we have a number of data properties to specify various number-, string- and URI-valued attributes of entities. In particular, *hasNormalizedForm* associates a point mutation code like “I615S”, with a point mutation instance, and *hasSequence* links a protein instance to a string which is a FASTA representation of the protein’s amino acid sequence.

### Use cases

Here we introduce the use cases we have adopted to test the suitability of SADI as a medium for providing access to our Mutation Impact software and data. All our use cases are in the form of queries, i.e., the user is seeking some information from publications or our Mutation Impact DB, in combination with external resources.

**Use case 1: Given a list of publications, identify mutations studied in the papers with their wildtype proteins and impacts on protein properties.** In this scenario, a biologist wants a quick summary of mutations studied in a set of papers. He is specifically interested in the proteins being studied as well as the identified change of protein properties. This kind of summarisation can aid literature search in many practical settings, e.g., when a biology researcher looks for related work for a publication. It can also be used by bioinformatics database curators to populate or verify databases.

**Use case 2: Find all mutations and the structure images of wild type proteins that were mutated, where the impact of the mutation is an enhanced haloalkane dehalogenase activity.** In this use case we aim to address the needs of a protein engineer who is seeking to understand what mutational changes can enhance the catalytic activity of an industrial enzyme, which is haloalkane dehalogenase in this scenario. The medium for reviewing the causal relationship of mutations on protein activity is a protein structure image which can be annotated with mutations and their impacts retrieved from a database/triplestore [22] or extracted automatically from documents using text mining techniques [5,23]. In our use case, we perform retrieval of the specific protein structures where there are published reports of mutations having a positive impact on catalytic activity. The user would wish to retrieve and review these structures along with mutation locations and impact annotations. The expected output of the integrated SADI services is the selected protein structure files and the corresponding mutations. Ideally, we would like to see the amino acids in the mutation positions highlighted on the 3D image of the protein, as it is done in mSTRAP [5].

**Use case 3: Find all pathways, together with the corresponding pathway images, that might have been altered by a mutation of the protein Fibroblast growth factor receptor 3.** In this scenario we address the needs of a systems biologist who is seeking to understand the likely impact of reported mutations on signalling or metabolic pathways [24] in which the mutated protein participates. This entails the retrieval of pathway information for the mutated proteins, which can be provided as a pathway diagram also. In the current use case we deal with mutations to the protein *Fibroblast growth factor receptor 3* reported in scientific papers which impact the protein either positively or negatively.

**Use case 4: Find all drugs related to mutated proteins, together with their interaction partners, where the mutation impact is a decreased carbonic anhydrase activity.** In this use case we address a query that a researcher in drug discovery would make when looking for existing drugs targeting a new disease condition. In the case of Carbonic anhydrase, an enzyme involved in the acid-base balance of blood (via the interconversion of carbon dioxide and bicarbonate), enzyme inhibitors such as acetazolamide, cause mild metabolic acidosis. This can be beneficial to patients with severe chronic obstructive pulmonary disease (COPD) with chronic hypercapnic ventilatory failure who need a reduction in arterial carbon dioxide and a rise in arterial oxygen and the transport of carbon dioxide out of tissues. The query will help us to identify the names of known drugs targeting the enzyme and what experimental modifications on the protein have resulted in lowering its activity *in situ*. Moreover, the query will also retrieve the names of proteins that interact with the enzyme directly through protein-protein interactions.

**Use case 5: From the literature, find all reported mutations of the protein with the nsSNP rs2305178.** In this use case, a researcher in genomics asks for all known mutations reported in the literature for a protein containing the non-synonymous SNP identified with the dbSNP ID rs2305178. By retrieving all known mutations for the protein in which the nsSNP is reported, the researcher can find out if any of these reported mutations corresponds to the location of the SNP in question. Minimally, the researcher can retrieve the full set of mutations to the protein based on reported experimental analysis and their impacts, together with references to the supporting literature. In our settings, we assume that the scope of the search is limited to the publications that have been processed with our text-mining software and semantically indexed in our Mutation Impact DB.

## Results

### SADI services for Mutation Impact pipeline and data

As an initial implementation with SADI, we created a service that takes a text in the form of a string literal or, alternatively, a URL of a file with the text, and outputs all property assertions derived from the input text, such as links from the text identifier (URI) to the extracted grounded mutations. These grounded mutations also have links to ungrounded mutations, proteins and impacts, in their descriptions. The main purpose of this service is to provide programming- and installation-free access to our text mining pipeline. In fact, we currently use this service ourselves to populate the Mutation Impact DB with OWL ABox assertions, because it has the capability of converting the raw results of the Mutation Impact pipeline to OWL. The service can also be useful in combination with services that find documents that have to be subsequently analysed.

We illustrate the operation of the service with the following example. In the simplified definition of the input class (in Manchester Syntax [15]) given below, individuals eligible as input to the service are required to be instances of *bibo*:*Document*, have their string content attached with the predicate *bibo*:*content* and to have the MIME type “text/plain” attached with *dc*:*format*:

The output class definition indicates that the service will attach instances of *mio*:*MutationSpecification* to the input URIs via the predicate *foaf*:*topic*:

We also provide an extract from the definition of the class *MutationSpecification* in the mutation impact ontology, that specifies how the wildtype protein, series of point mutations and corresponding impact are associated with a mutation specification instance:

Here is a sample input to the text-mining service:

Note that the value of *bibo*:*content* is a string with the ASCII content of the article with PubMed Central ID 100293 represented with the URI *pmc*:100293.

This RDF listing shows the corresponding output of the service:

Most of our other mutation impact-related SADI services essentially wrap some ad hoc queries to our Mutation Impact DB. For example, one of the most intensively used services – *getMutationByWildtypeProtein* – finds all instances of the Mutation Impact ontology class *MutationSpecification*, given the UniProt ID of a protein that acts as the wildtype protein in those mutations. More specifically, the service expects an RDF node, representing a protein, with a UniProt record attached to it via *sio*:*SIO_*000212 (’is referred to by’), which is in turn linked via *sio*:*SIO*_00000*8* (’has attribute’) to an attribute of the type *lsrn*:*UniProt-Identifier*, whose string value is attached to it with *sio*:*SIO_*000*3*00 (’has value’). This listing provides a simplified version of the input class:

This kind of input modelling makes the service semantically interoperable with many other SADI services working with proteins.

In the output, the service attaches a mutation specification instance to the protein via the predicate *mio*:*proteinIsSpecifiedAsWildtypeBy*, which is an inverse of *mio:groundMutationsTo.* The class *MutationSpecification* is central to the ontology and the DB: its instances represent grounded mentions of mutations and are linked to the corresponding wildtype and mutant proteins, the mutation impacts, and also the texts from which the mutation mentions were extracted. So, two other services - *getMutationByMutantProtein* and *getMutationByImpact* - also find *MutationSpecification* instances by their mutant proteins and required mutation impacts.

Two other services retrieve instances of biological entities of specified types, present in our DB. The service *getMIDBBioEntityByType* does this for the top level biological entity classes in our ontology, such as *Protein* or *Point Mutation.* The service *getProteinPropertyByType* specialises in protein property types, most of which are currently inherited from the Gene Ontology. Given a subclass of *ProteinProperty*, e.g., *GO*_0018786 (’haloalkane dehalogenase activity’) from the Gene Ontology, it finds all known instances of this type, whose descriptions contain links to the proteins they characterise.

There are also two auxiliary services: *getMutationImpactByProteinProperty* finds mutation impact instances linked to a specified protein property grounded to a specific protein, and *getMutationSubseries* finds series of elementary mutations identified in a text, that are subsets of a specified set of elementary mutations. We also have two services that visualise grounded mutations by rendering the 3D structure of the wildtype proteins and highlighting the amino acids affected by the point mutations.

The list of all SADI services based on the Mutation Impact ontology, text mining pipeline and DB, can be found in [25] and is also summarised in Table 2.

**Table 2:** Our SADI services based on the Mutation Impact ontology, text-mining pipeline and database. Detailed information (in RDF) about a service can be obtained by opening the service URL, obtained by attaching the prefix http://unbsj.biordf.net/mi-sadi/ to the name, in a Web browser.

service	operation
*mineTextForMutationImpacts*	extracts mutation specifications from a document
*getMutationByWildtypeProtein*	finds specifications of mutations grounded to a given protein
getMutationByMutantProtein	finds specifications of mutations resulting in a protein specified by its sequence
*getMutationImpactByProteinProperty*	finds mutation impact instances affecting a specified grounded protein property
*getMutationByImpact*	finds mutation specifications corresponding to an impact on a specified grounded protein property
*getMutationSubseries*	finds mutation series instances that are subseries of a given mutation series
*getMIDBBioEntityByType*	finds biological entities by their type URIs
*getProteinPropertyByType*	finds protein properties grounded to specific proteins by their type URIs
*visualiseMutationSeries*	renders the 3D structure of the wildtype protein, from PDB, and highlights the point mutation positions
*visualiseMutationSeriesWithHomologyModeling*	same as visualiseMutationSeries except that the 3D structure is predicted by homology modeling

### Experiments with SHARE

This section contains the main result of our investigation – it describes our experiences using SADI via the SPARQL engine SHARE to solve the use cases.

In the query examples below we omit prefix declarations – the meaning of the namespace abbreviations is given in Table 1. Full versions of all queries discussed in this article are available from [18], with instructions on how to execute them via a SHARE Web interface installed locally for this purpose.

#### Experiment with use case 1

In this use case, our goal is to formalise the query “*Given a list of publications*, *identify mutations studied in the papers with their wildtype proteins and impacts on protein properties*” and execute it using our text mining pipeline for mutation impacts. We have uploaded three PDF files with publications about mutations to a location on the Web and listed their URLs in an RDF document (http://unbsj.biordf.net/util-sadi-services/service-data/PDFs.rdf) that will serve as input to our SPARQL query. This document describes the files as instances of the class *bibo*:*Document* having the MIME type “application/pdf” as the value of the *dc*:*format* predicate. For example, the paper with the PubMed ID 17545153, uploaded to our Web site, is represented with the following entry, given, for readability, in the Notation 3 syntax [16] for RDF:

In general, we often need to create such RDF documents to specify input to queries or to provide additional information necessary to execute the queries, because SPARQL does not allow inlining assertions in queries directly.

We start with the following simple SPARQL query:

where http://unbsj.biordf.net/.../PDFs.rdf abbreviates http://unbsj.biordf.net/util-sadi-services/service-data/PDFs.rdf.

The purpose of this query is essentially to list mutation specification instances (?*MutationSpec*) together with the input documents (?*PDF Document*) they are extracted from. Our text mining SADI service provides the predicate *foaf:topic.* However, writing a condition like ?*PDF Document foaf:topic* ?*MutationSpec* is not enough because the service only accepts documents in ASCII, whereas our input documents are in PDF. Moreover, we are modelling a situation where *the user does not know what text formats are accepted by the available text mining services.* So, line 5 requests a conversion of ?*PDF Document* into all available formats: the predicate *dc*:*hasFormat* relates different representations of the same document and is provided by our SADI service *pdf*2*ascii.* Finally, line 4 is needed to enumerate PDF documents from the input. Note the use of *dc*:*format* to specify the MIME type of a document.

The query executes in less than one minute and returns twenty six mutation specifications extracted from the three papers from the input file *PDFs.rdf.* However, returning only mutation specification instances like *mio*:*MutationSpecification*1292519446381_2538 is clearly not enough. Our imagined user needs various informative parts of a mutation specification, such as the wildtype protein and identified impact, rather than just a URI. In the service output, these are attached with various predicates, such as *mio*:*groundMutationsTo* or *mio*:*specifiesImpact*, and can be easily requested in the query by adding the following lines:

Line 1 extracts the reference to the wildtype protein. Lines 2-4 extract codes like “I615S” for all the point mutations referenced by the mutation specification. Line 5 extracts the impact instance, line 8 extracts the direction, e.g., *mio*:*Positive* or *mio*:*Neutral*, assigned to the impact instance, and lines 6-7 extract the types of the affected protein property, e.g., *go*:*GO*_0004016. The SELECT line in the new query can specify ?*PDF Document*, ?*Protein*, ?*NormalizedMutation*, ?*ImpactDirection* and ?*ProteinPropertyType* as the answer variables, so the user now can see answers like this:

The actual answer is given by SHARE in the form of a table where the columns are labelled with the query variables. We do not show the table here as it does not fit due to very long rows. Note also that there may be multiple rows with the same wildtype protein but different point mutations or affected protein properties.

**Figure 2:**
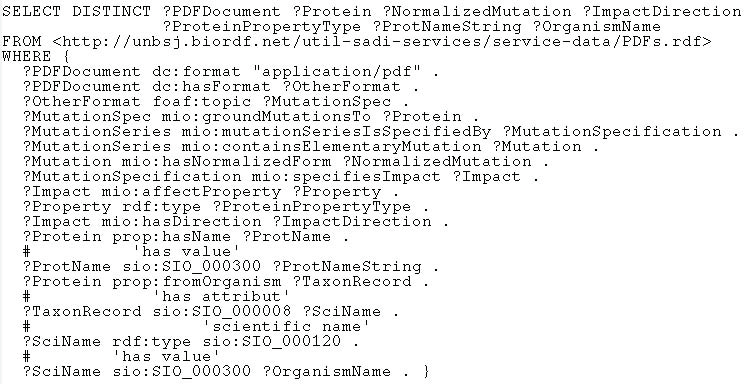
**Listing of the final SPARQL query for use case 1.** This SPARQL formalises “Given a list of publications, identify mutations studied in the papers with their wildtype proteins and impacts on protein properties”.

Although such results are already satisfactory, for extra user convenience we would like to provide readable protein names and the organisms they belong to, in addition to the UniProt IDs like “O75907”. None of our services can deliver this information, so we look in the central SADI registry [19] for appropriate predicates and find *prop*:*hasName* that relates a protein (UniProt record) to an attribute representing the name of the protein, whose string value is accessible via the data property *sio*:*SIO_*000300*.* There is also predicate *prop*:*fromOrganism* relating a protein to the corresponding taxon record that is linked to its scientific name attribute via *sio*:*SIO_*000008*.* Both predicates are provided by the service *uniprotInfo* we found in the public registry [19]. The listing for the final query is given in Figure 2. In about three minutes, the execution of this query produced several dozens of bindings like the following one:

The main message we would like this use case to deliver is that by packaging our text mining software as a SADI service we offer its functionality to the end users in a programming-free manner. This possibility alone already makes SADI a valuable part of our infrastructure for annotating mutations. The use of a separate service for PDF-to-ASCII conversion demonstrates the extra flexibility this approach provides - one can use our text mining service with any text formats, provided that there are SADI services extracting ASCII contents from these formats. Note also how easy was it to present our text mining results in combination with data from external sources, as exemplified by the use of the *uniprotInfo* service. In the next four use cases we will focus our attention on the value of such integration.

#### Experiment with use case 2

**Figure 3:**
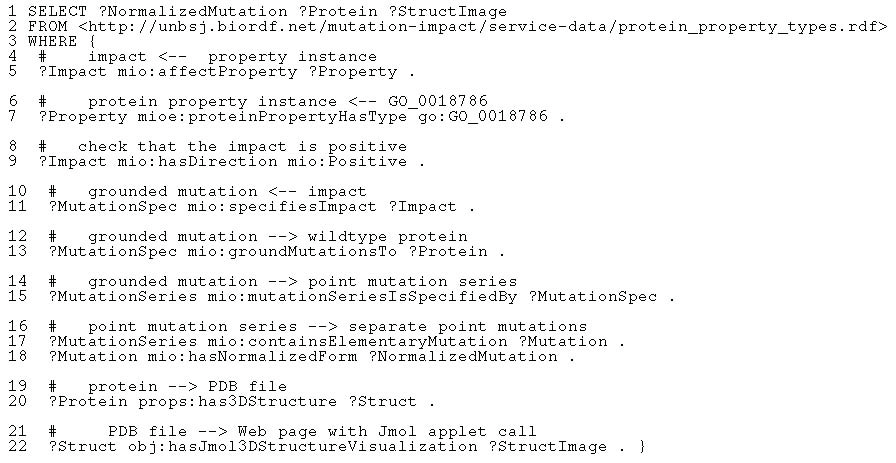
**Listing of the baseline SPARQL query for use case 2**. This SPARQL formalises “Find all mutations and the structure images of wild type proteins that were mutated, where the impact of the mutation is an enhanced haloalkane dehalogenase activity”.

**Baseline functionality: show the protein structure.** Our query *“Find all mutations and the structure images of wild type proteins that were mutated*, *where the impact of the mutation is an enhanced haloalkane dehalogenase activity”* can be realised with the SPARQL shown in Figure 3. Let us analyse how we construct this query. The predicate *mioe*:*proteinPropertyHasType* in our ontology, provided by the service *getProteinPropertyByType*, links grounded protein properties with their types, so we can use it to enumerate known instances of *GO*_0018786. In lines 5 and 9, *mio*:*af fectProperty* links the grounded protein properties to the corresponding instances of mutation impacts and *mio*:*hasDirection* selects only positive impacts. Using *mio*:*specifiesImpact*, we can select instances of mutation specifications (line 11), which in turn link to the corresponding wildtype proteins (line 13) and series of elementary mutations (line 15). We would like to see readable codes of elementary mutations in the output, like D124N or V226A, so we use *mio*:*containsElementaryMutation* to retrieve the corresponding elementary mutations and *mio*:*hasNormalizedForm* to map them to the corresponding codes.

So far we have used only predicates from our Mutation Impact ontology. Since the essence of use case 2 is visualisation, we look for predicates in SADI-related ontologies, that could link proteins to their images. There is no direct link, but we can use the composition of *props*:*has3DStructure* and *obj*:*hasJmol3DStructureVisualization* to first retrieve a reference to the PDB record of the protein, and then find the corresponding graphics file.

SHARE was able to compute our query using three of our SADI services – *getProteinPropertyByType*, *getMutationImpactByProteinProperty* and *getMutationByImpact* – and two third party SADI services from the registry, providing *props*:*has3DStructure* and *obj*:*hasJmol3DStructureVisualization*, and yet this was completely transparent to us as the end users. We only dealt with an almost completely declarative query composed of predicates that we were able to find in ontologies referenced by available SADI services. The only thing we need to know beyond the semantics of a predicate is the direction in which available services compute it: e.g., we cannot use *props*:*has3DStructure* to get from a PDB ID to the corresponding protein because there is currently no service that would annotate a PDB ID with the inverse of *props*:*has3DStructure*. Finding the services, their invocation and some deduction with the ontological definitions of predicates, was done by SHARE completely automatically. Note especially the ease with which integrating our mutation-related information with the external sources of data was achieved.

**Figure 4:**
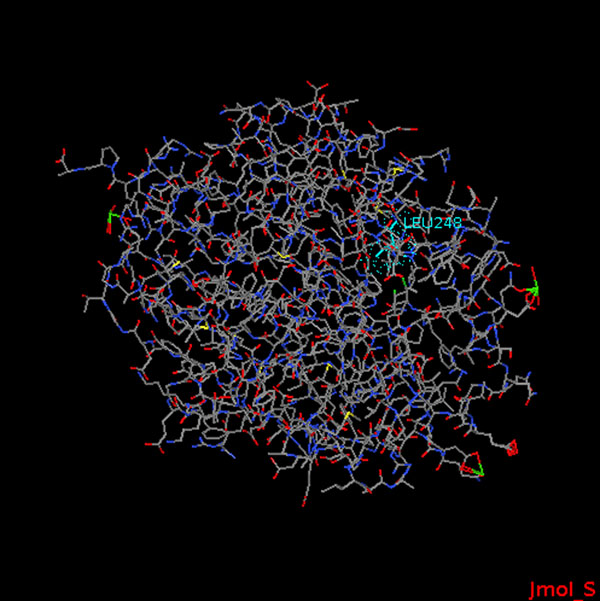
**Screenshot of a Jmol rendering of the structure of P51698 with L248I.** This image was obtained by running the Jmol viewer on a PDB file representing the amino acid sequence of protein with the UniProt ID P51698. The highlighted amino acid is the wildtype of the point mutation L248I.

**Extended functionality: locating mutations on the protein structures.** Although the query above illustrates well the integrative power of SADI and SHARE, it does not fully satisfy the requirements for the use case because the mutations are not shown on the protein 3D structure. At the time of our experiments, no existing SADI services were providing such functionality, so we wrote our own service *visualiseMutationSeries*. This service accepts a mutation specification including a protein instance identified with a UniProt record URI, as input. It extracts references to PDB [26] files representing parts of the protein sequence obtained by different methods, e.g., X-ray crystallography, from the UniProt record. Then it creates a small Jmol [27] script that instructs Jmol to render the amino acid sequence with the positions of the specified point mutations highlighted on the structure. In the output, the service links the input mutation specification to an HTML document using the predicate *obj*:*hasJmol3DStructureVisualization*. This small HTML document calls the Jmol viewer applet on the created script, so that when it is loaded into a Web browser with Java applet support, the user can see and rotate the 3D image of the protein structure with wildtype residues highlighted on it. Figure 4 is a screenshot of a Jmol rendering of the structure of P51698 with the wildtype residue of the point mutation L248I highlighted.

**Figure 5:**
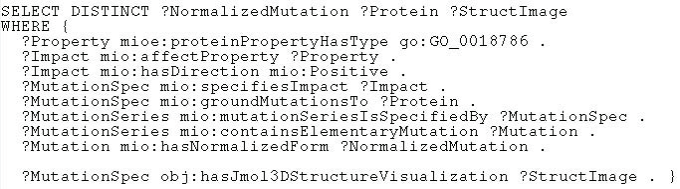
**Listing of the extended functionality query for use case 2**. Improves on the query in Figure 4 by requesting mutations to be shown on the protein 3D structure.

All it takes to use the *visualiseMutationSeries* service for the purposes of our use case is to replace lines 19-22 with the triple pattern

as shown in Figure 5.

**Homology modelling for missing structures.** Our experiments with mutation visualisation using the known protein structures from the Protein Data Bank (PDB) [26] revealed that many proteins of interest don’t yet have PDB records. To rectify this, at least partially, we adopted the solution used in mSTRAPviz [5]. If the amino acid sequence of a protein is known, which is usually the case with UniProt listed proteins, we look for homologous sequences for which PDB files exist and then call the MODELLER program [28] to predict the 3D structure of the target protein by adjusting the structures of the template sequences.

To implement this, we created the SADI service *visualiseMutationSeriesWithHomologyModeling* that takes a mutation specification with a wildtype protein whose amino acid sequence is given as a FASTA string, as input. The protein’s sequence must also have a homologue identified by a PDB record. The service runs MODELLER on these data and the created PDB file representing the predicted structure is treated exactly the same way as *visualiseMutationSeries* treats files hosted by the Protein Data Bank, i.e., it is visualised with Jmol, together with the specified point mutations. Additionally, we have written the SADI service *blastPDB* that wraps a PDB SOAP service based on the BLAST algorithm for searching for homologous sequences in the PDB database. To test the new services, we ran a query obtained by replacing *GO*_0018786 in the query in Figure 5, with *GO*_0004091, and requesting negative impacts, so that the relevant proteins in our Mutation Impact DB don’t have PDB files (details are provided in [18]). The query is executed in two minutes and returns visualisations of one protein *Esterase YpfH* with four distinct point mutations. Since two homologous PDB sequences are used to model the protein’s 3D structure, the total number of answers for the query is eight.

#### Experiment with use case 3

The work required by this use case (“*Find all pathways*, *together with the corresponding pathway images*, *that might have been altered by a mutation of the protein Fibroblast growth factor receptor 3“*) can also be divided into two parts: the first part can be done using the predicates from our ontology, and the second part has to be delegated to external resources, dealing with genes, pathways and pathway visualisation. Since we know that the wildtype protein is *Fibroblast growth factor receptor 3* (UniProt ID P22607), we can easily retrieve the mutation specifications linked to this protein with the property *mio*:*groundMutationsTo.* These instances will have impacts attached to them with *mio*:*specifiesImpact*, and we can specify the interesting impact directions with *mio*:*hasDirection.*

**Figure 6:**
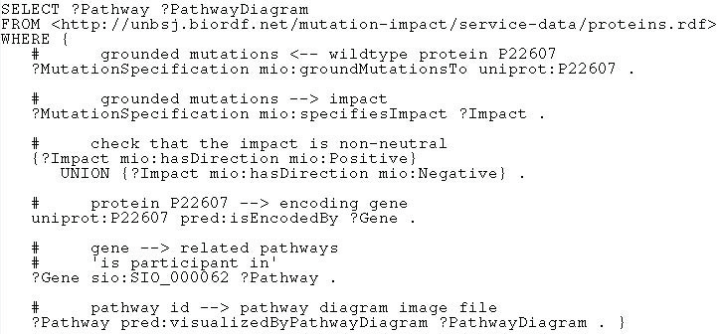
**Listing of the SPARQL query for use case 3**. This SPARQL formalises “Find all pathways, together with the corresponding pathway images, that might have been altered by a mutation of the protein Fibroblast growth factor receptor 3”.

Using *pred*:*isEncodedBy* we also map the protein to the corresponding gene, and *sio*:*SIO_*000062 (’is participant in’) allows to retrieve the pathways in which the protein participates, *pred*:*visualizedByPathwayDiagram* will fetch the corresponding graphics file URL. The resulting query is shown in Figure 6. Note that the input file in the FROM clause just qualifies *uniprot*:*P*22607 as an instance of *mio*:*Protein* to make it a legitimate input to the service *getMutationByWildtypeProtein* that links proteins to mutations specifications. SHARE executed the query using this service and two external SADI services providing *sio*:*SIO_*000062 and *pred*:*visualizedByPathwayDiagram.* The execution took less than one minute and returned five pathways with diagrams.

#### Experiment with use case 4

**Figure 7:**
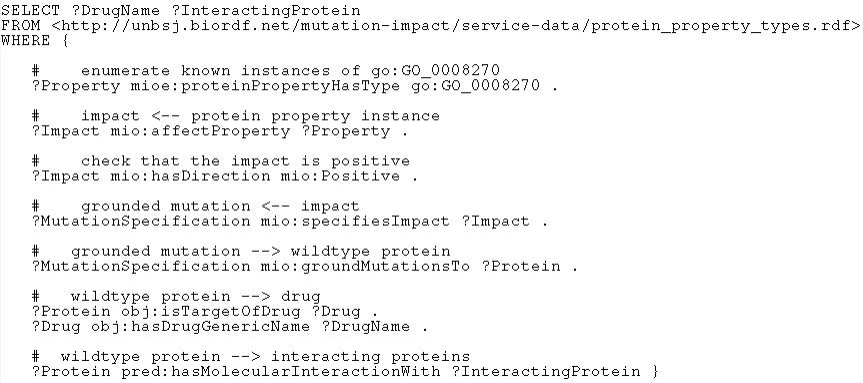
**Listing of the SPARQL query for use case 4**. This SPARQL formalises “Find all drugs related to mutated proteins, together with their interaction partners, where the mutation impact is a decreased carbonic anhydrase activity”.

This use case (“*Find all drugs related to mutated proteins*, *together with their interaction partners*, *where the mutation impact is a decreased carbonic anhydrase activity*”) is somewhat similar to use case 2: given the protein property type, we retrieve the grounded properties, positive impacts and the wildtype proteins with the help of some predicates from our ontology. The connection from the proteins to drug names is realised with the predicates *obj*:*isTargetOfDrug* and *obj*:*hasDrugGenericName.* Separately, we find the interacting proteins with *pred*:*hasMolecularInteractionWith.* To make *go*:*GO*_0008270 a valid input to our service *getMutationImpactByProteinProperty*, it is qualified as a *mioe*:*ProteinPropertyType* in the input file in the FROM clause. The resulting query is shown in Figure 7. The query was executed in less than two minutes and returned 50 distinct drug names and 2 interacting proteins.

#### Experiment with use case 5

**Figure 8:**
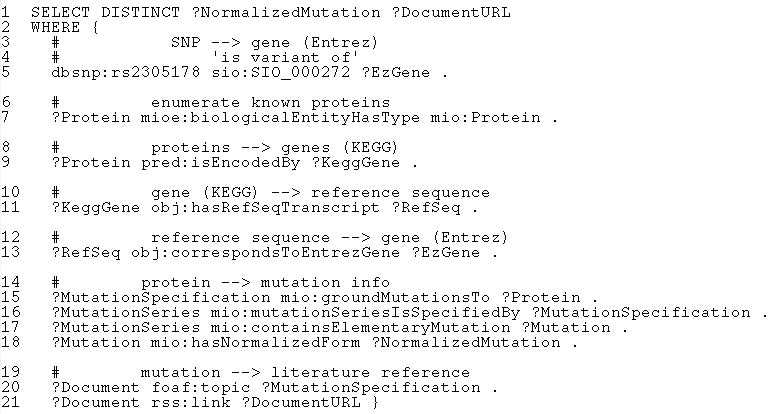
**Listing of the SPARQL query for use case 5**. This SPARQL formalises “From the literature find all reported mutations of the protein with the nsSNP rs2305178”.

Finally, the query “*From the literature find all reported mutations of the protein with the nsSNP rs2305178*” was implemented with the SPARQL query shown in Figure 8. The predicate *sio*:*SIO_*000272 (’is variant of) in line 5 maps the specified dbSNP ID to an Entrez gene ID. If we were dealing with completely declarative queries, it would be enough to use a composition of the predicates *obj*:*correspondsToEntrezGene*, *obj*:*hasRefSeqTranscript* and *pred*:*isEncodedBy*, as in lines 9-13, to map the Entrez gene ID to a protein. However, no SADI service currently provides the inverses to the first two predicates, so the composition can only work in the direction from proteins to Entrez gene IDs. To use this possibility, we had to implement the service *getMIDBBioEntityByType* that enumerates all proteins known in our DB. In fact, the service is more general - it enumerates instances of several main biological entity classes from our ontology, such as *MutationImpact* or *PointMutation.* The service provides the inverse of *mioe*:*biologicalEntityHasType* whose use is demonstrated in line 7. Linking the protein to elementary mutations is done exactly the same way as in use case 2. Once SHARE has the necessary data in the working memory, it computes the join on the variable ?*EzGene.* Finally, the last two lines in the query serve to retrieve the URLs of the documents from which the corresponding mutation specifications were extracted.

## Discussion

We are not aware of any work solving exactly the same problem, i. e. publishing text-mined information on mutations and text-mining software itself, with semantic web services, so we look at related work falling into a more general topic. Since the problem we are solving is essentially an instance of the more general problem of agile integration of bioinformatics resources with the help of semantic web services, we will refer the reader to two projects in this area.

BioMOBY [29] is the most closely related technology, simply because it is a direct predecessor of SADI - the SADI project emerged as an attempt to better integrate services into the general Semantic Web context [11]. SADI inherited much of the BioMOBY ideology, in particular that the messages exchanged between clients and services carry their semantics by using ontology-based formats, and the decentralised domain ontology use. From the perspective of our case study, the key advantage of SADI is that the relation between inputs and outputs is explicitly ontologically defined, whereas BioMOBY still follows the earlier semantic web service paradigm that only requires a service’s functionality to be *categorised*, e. g. by qualifying the service as an instance of an ontological class, say *SequenceAllignmentService*, and by mapping the input and output data types to ontological classes, possibly from a domain ontology. This difference makes us strongly prefer SADI because all our use scenarios assume, as a user, a biologist rather than a bioinformatician who would be comfortable with an ontology of bioinformatics operations and data types. We also assume that in many cases a non-bioinformatician user will also prefer dealing with declarative queries that are executed completely automatically, to creating workflows, even with the help of tools that exploit the service categorisation and the semantics of service IO to ease such workflow creation.

Both SADI and BioMOBY require service providers to adhere to the IO conventions imposed by these frameworks. However, access to many bioinformatics resources is already available in the form of Web services consuming and producing ad hoc XML-based formats, e.g., SOAP services. Such legacy services, as well as new Web services whose providers cannot or don’t want to make them natively semantic, can sometimes be turned into Semantic Web services by *semantic annotation*. my-GRID [30,31] is a mature project that follows this approach by allowing services described with WSDL to be annotated, possibly by a third party, with respect to a centralised ontology. Although the use of unrestricted XML as the data model for service IO is a great convenience, some other features of myGRID make its use for our purposes problematic. First, as in the case with BioMOBY, there is no way to describe the semantics of a service by ontologically relating the input and output. Second, the necessity of conversions between datatypes consumed and produced by different services seems to complicate the workflow construction – this gives services “speaking” the same language a clear advantage. Finally, the reliance on a centrally curated ontology would deprive us of the extra flexibility in semantic modelling of services that the SADI and BioMOBY approaches enjoy. In the concrete settings of our case study, it is unclear how we could substitute the classes and predicates from our Mutation Impact ontology with terms from, for example, the myGRID Domain Ontology.

## Conclusions

The primary goal of our case study was to explore the suitability of the SADI framework as a medium to facilitate data sharing and integration across biological data types. We have identified that SADI provides an effective way of exposing our mutation impact data such that it can be leveraged by a variety of stakeholders in multiple use cases.

Our experience in deploying and registering mutation services in accordance with SADI specifications was positive, albeit with some challenges. In particular, we identified that advanced skills in knowledge engineering were required to build semantic representations of the services. More specifically, a SADI service provider has to (i) find classes and predicates in existing ontologies, that model his data well, and (ii) ensure that his modelling of service IO is compatible with the IO of other SADI services with which the new service is intended to be composed. The first task is a general problem for all activities requiring ontology-based modelling, and seems to have no simple solution. It seems safe to assume that at least in the near future this task has to be performed mostly manually by reasonably experienced knowledge engineers. Difficulties associated with the second task are likely to be alleviated with the appearance of more sophisticated tools for browsing networks of SADI services.

We also note that formulating the queries based on the SADI services requires cumbersome search for predicates in the SADI-related ontologies. Clearly, the necessary infrastructure for such search is yet to be built.

Another conclusion we have drawn from our case study is that a greater choice of available SADI clients is necessary to make SADI practically useful, especially in production settings. We will look at the SADI plugin for Taverna [32], which is currently under active development.

Most, if not all, of our queries could be replaced with browsing, especially faceted, of the virtual RDF graph implied by the services, which is much more user friendly than writing SPARQL queries. Unfortunately, the only currently available RDF browser with SADI support is Sentient Knowledge Explorer (see, e.g., [13]), which is a commercial product.

Another important conclusion we have drawn from our experiments is that some limitations of the SADI-based approach to data integration also restrict its applicability strictly to the *discovery phase* in a scientific or R&D process. In simple words, one can use SADI to come up with hypotheses and obtain preliminary evidence, but SADI-produced results cannot be used as hard evidence. The relevant limitations are the *absence of answer completeness guarantee* with the existing query client, *absence of result reproducibility guarantee* and *lack of answer justifications*. The absence of completeness guarantee, mentioned in the section about SHARE, and the inherent irreproducibility of results due to the reliance on third party services that can be down, inaccessible, etc., make statistical judgements based on answers returned by SHARE unreliable, although some valuable insights can be obtained and used later to drive more rigorous investigations. Creating clients that would provide verifiable answer justifications seems a good target for research.

The fact that the initial query design for Use case 5 did not work because some services were missing suggests that the general utility of SADI is predicated on the coverage of bioinformatics resources and relevant onto-logical predicates by existing services. In this respect, we would like to mention that the SADI network of public services is growing fast – it is expected to contain over 400 services by the end of 2011.

In future work we aim to extend the Mutation Impact DB with more data types related to mutation annotations extracted from the literature, and create the corresponding SADI services facilitating integration with other Bioinformatics data. We are also conducting case studies on the use of SADI for other biomedical domains, such as lipidomics and experimental proteomics data.

Apart from the integration of distributed and heterogeneous sources of data, the SADI framework can be useful simply as a medium for *semantic querying* of a single database, so that SPARQL queries can be answered on an SQL database. We are exploring this possibility in a case study with a large health care research datawarehouse.

## List of abbreviations used

GATE: General Architecture for Text Engineering; SADI: Semantic Automated Discovery and Integration; SPARQL: SPARQL Protocol and RDF Query Language; SHARE: Semantic Health and Research Environment; GO: Gene Ontology; OWL: Web Ontology Language; RDF: Resource Description Framework; URI: Universal Resource Identifier; SIO: Semanticscience Integrated Ontology; FOAF: Friend-of-a-friend ontology; SNP: single-nucleotide polymorphism; MIME: Multipurpose Internet Mail Extensions; PDB: Protein Data Bank; SOAP: Simple Object Access Protocol.

## Competing interests

The authors declare that they have no competing interests.

## Authors’ contributions

AR wrote the SADI services and did the experiments with the SPARQL queries. JBL co-developed the use cases with CJOB and contributed to the SHARE experiments at the early stages. CJOB coordinated the work. All authors contributed to the manuscript.
